# Mathematical Model of HPV Provides Insight into Impacts of Risk Factors and Vaccine

**DOI:** 10.1371/journal.pmed.0030164

**Published:** 2006-04-04

**Authors:** Jane J Kim

## Abstract

Kim discusses a study by Barnabas and colleagues in *PLoS Medicine* in which the researchers used a mathematical model to assess the effect of sexual transmission of HPV 16 in a Finnish population.

Over the next few years, we can expect to see the licensing of one or more prophylactic vaccines against human papillomavirus (HPV) infection, considered a necessary precursor to cervical cancer. Among over 40 known types of HPV, 15 have been identified as being “high risk” or oncogenic. The vaccines will likely target at least two of these oncogenic types, HPV types 16 and 18, which have been found to contribute to nearly 70% of cervical cancer cases worldwide—although there is large variation in contribution by geographic region [ 1, 2]. Although cytology-based cervical cancer screening has been successful in decreasing the incidence of invasive cancer in many developed countries, vaccination may be the most promising intervention from a global perspective—reducing the burden of cervical cancer in resource-poor settings, where most cases occur.

## Why a Mathematical Model?

Mathematical models can be useful tools in exploring disease trends and health consequences of interventions in a population over time. In the case of cervical cancer, in which the time from acquisition of HPV infection to development of invasive cancer can be two decades or more, models can be used to translate short-term findings from vaccine trials into predictions of long-term health outcomes [ 3–9]. In a new study in *PLoS Medicine*, Barnabas and colleagues present a particular type of mathematical model known as a dynamic model to directly assess the effect of sexual transmission of HPV type 16 in a Finnish population [ 10]. This model is the first to capitalize on empirical data on sexual history and HPV seroprevalence from a single population to estimate the transmission of HPV 16 between men and women, and to explore the impacts of risk factors on observed changes in cervical cancer incidence over time.

## What Did the Researchers Do?

The model itself, although technically complicated, is conceptually straightforward when broken down into its vital parts. Adult men and women were modeled separately, and in the model they formed partnerships over time based on sexual history data from Finnish adults within the period of 1971–1999. HPV type 16 is transmitted between men and women in sexual relationships at a rate that is dependent on the prevalence of HPV 16 in the population, as well as on age and sexual activity level; women infected with HPV 16 can further develop precancerous lesions and invasive cervical cancer (ICC).

Models can be used to translate short-term findings from vaccine trials into predictions of long-term health outcomes.

As a general rule, not all parameters in a model are known, which requires adjustment (or “calibration”) of model inputs to observed outcomes. One of the biggest unknowns in Barnabas and colleagues' model was the transmission rate of HPV 16 between partners. Since we cannot observe transmission per partnership directly, the authors calibrated this parameter using a maximum likelihood–based approach, which, in simple terms, derives an estimate of this parameter that achieves the best model fit to the observed HPV seroprevalence data from Finnish women. Borrowing other model inputs from previously published models [ 11, 12], the authors used their model to examine the contributions of risk factors (i.e., age at sexual debut, number of sexual partners, and smoking patterns) on the observed increase in ICC among 35- to 39-year-old women over time. They also used the model to consider a series of “what if?” scenarios pertaining to vaccination against HPV 16 on incidence of HPV 16–associated ICC.

## The Study's Key Findings

In estimating the transmission rate of HPV 16 between men and women, the authors found that the model underestimated the empirically observed seroprevalence of HPV 16 when they assumed the same rate of partner change as reported in the Finnish surveys. Consequently, they explored the effects on the model of increasing the number of new partners per year, and derived a transmission rate of 0.6 per partner after doubling the reported rate of sexual partner change, and 0.4 after assuming that the number of reported lifetime partners is actually the number of new partners per year.

Neither changes in sexual behavior nor smoking patterns were found to contribute substantially to the rising trends in HPV 16–associated cervical cancer over time for 35- to 39- year-old women. But the authors assumed only a one-time shift in age of sexual debut and in number of partners in the model, and assumed that smoking patterns of women were relatively constant from the late 1970s through the mid-1990s, as suggested by the empirical data. The authors note that other risk factors such as oral contraceptive use and parity may contribute to the changing patterns of incidence over time, but these were not included in the model.

For the most part, the analyses evaluating vaccination policies showed intuitive results: vaccination of both men and women has a small incremental benefit compared with vaccination of women alone; vaccine benefit decreases as age at vaccination—after sexual debut—increases; and a shorter duration of vaccine-induced immunity is shown to be associated with higher cancer incidence, particularly if waning immunity coincides with higher rates of persistent infections among older women. When vaccine protection is assumed to be lifelong, cytology-based screening is predicted to add little benefit to vaccination alone, but the outcome reported is for ICC associated with HPV 16 only. It is important to bear in mind that even under the most favorable conditions of vaccine coverage, efficacy, and duration, some form of screening will likely be necessary to reduce ICC associated with the 13 or more other oncogenic HPV types not targeted by the vaccine.

## Study Limitations and Implications

This model represents an impressive step forward in the simulation of HPV transmission and in the exploration of the effects of risk factors and vaccination over time. However, the conclusions drawn from this analysis should be interpreted with an appreciation of the model's limitations. Most obviously, the model in its current form reflects HPV infection and cervical cancer associated with only a single HPV type. Therefore, the model cannot provide a comprehensive picture of cervical cancer over time after the introduction of vaccination; indeed, another 30% of cervical cancer cases (and more in some regions of the world) are caused by oncogenic HPV types other than type 16. And while the potential for cross-protection or cross-reactivity among HPV types seems plausible, this potential cannot be explored using a single-type HPV model such as this one.

Furthermore, the authors conclude that the transmission probability per partnership lies above 0.4, which is based on the extreme assumption that the number of new annual partners is equivalent to the number of reported lifetime partners. While there are known biases in sexual behavior survey data, this value of per-partner transmission relies on many other uncertain parameters and assumptions in the model. Assumptions such as lifelong acquired immunity, where women are no longer susceptible to repeat infections after initial infection and clearance, are acknowledged by the authors to affect the estimated HPV 16 transmission probability, but are not explored in the analysis. Holding all else constant, we would expect to see the transmission probability decrease below 0.4 if the assumption of lifelong acquired immunity is relaxed. Lastly, trends in sexual behavior as well as other risk factors will differ in other settings, meaning that Barnabas and colleagues' findings are not generalizable to other settings. The model itself, however, may be used for analyses in other countries, but preferably after a recalibration of the model using available country- or region-specific data.

We can anticipate better data on the longitudinal nature of HPV infections, including immunity and interactions among multiple types and even the transmission rate of HPV among partners. In the meantime, models such as the current one can be valuable tools for exploring the impacts of known risk factors in the cervical carcinogenesis pathway for a single HPV type and for gaining insights into hypothetical scenarios of efficacy, duration, and long-term impact of type-specific vaccination. 3

## Abbreviations

HPV: human papillomavirus
ICC: invasive cervical cancer
